# Association between organizational climate and perceptions and use of an innovation in Swedish primary health care: a prospective study of an implementation

**DOI:** 10.1186/s12913-015-1038-2

**Published:** 2015-09-10

**Authors:** Siw Carlfjord, Karin Festin

**Affiliations:** Department of Medical and Health Sciences, Division of Community Medicine, Linköping University, SE-581 83 Linköping, Sweden

## Abstract

**Background:**

There is a need for new knowledge regarding determinants of a successful implementation of new methods in health care. The role of a receptive context for change to support effective diffusion has been underlined, and could be studied by assessing the organizational climate. The aim of this study was to assess the association between organizational climate when a computer-based lifestyle intervention tool (CLT) was introduced in primary health care (PHC) and the implementation outcome in terms of how the tool was perceived and used after 2 years.

**Methods:**

The CLT was offered to 32 PHC units in Sweden, of which 22 units agreed to participate in the study. Before the introduction of the CLT, the creative climate at each participating unit was assessed. After 24 months, a follow-up questionnaire was distributed to the staff to assess how the CLT was perceived and how it was used. A question on the perceived need for the CLT was also included.

**Results:**

The units were divided into three groups according to the creative climate: high, medium and low. The main finding was that the units identified as having a positive creative climate demonstrated more frequent use and more positive perceptions regarding the new tool than those with the least positive creative climate. More positive perceptions were seen at both individual and unit levels.

**Conclusions:**

According to the results from this study there is an association between organizational climate at baseline and implementation outcome after 2 years when a tool for lifestyle intervention is introduced in PHC in Sweden. Further studies are needed before measurement of organizational climate at baseline can be recommended in order to predict implementation outcome.

## Background

Factors influencing the implementation of new methods in health care have been studied, but there is still a lack of knowledge regarding determinants of a successful implementation. The role of context has been highlighted, and is one essential part in the Promoting Action on Research Implementation in Health Services (PARIHS) framework; implementation success is considered to be a function of evidence, context and facilitation [1]. Cummings et al. [2] have described how contextual factors in terms of culture, leadership and evaluation have an impact on research uptake among nurses. Another contextual factor that has been suggested to influence the uptake of research findings is organizational climate, defined by Greenhalgh et al. [3] as the extent to which staff in an organization “feel that it’s OK to experiment with new ideas”. Organizational climate can be studied explicitly, or as one factor in what is often labelled as a receptive context [3]. The role of a receptive context for change to support effective diffusion of research evidence has also been underlined [4]. Another important factor associated with the potential adopters is whether they see a need for the innovation, meaning that it is perceived to have advantages compared with current practice [5]. However, effective implementation needs not only a receptive climate but also a good fit between what is implemented and the potential adopters’ needs and values [6]. This was considered when a computer-based tool for lifestyle intervention was developed and introduced in Swedish primary health care (PHC) [7].

In Sweden, health care has an obligation not only to provide medical care but also to address lifestyle issues in order to prevent illness and promote health [8]. Preventive services, however, are not provided to the fullest extent due to lack of time, knowledge and skills, a problem that has also been recognized in other parts of the world [9–11]. To meet the needs expressed by practitioners, a computer-based lifestyle intervention tool (CLT) was developed and offered to nine PHC units operating in Östergötland County in south east Sweden in 2006. The CLT was evaluated and found to be feasible for both staff and patients [7]. Adoption of the CLT was found to vary substantially between the PHC units and questions were raised about how contextual factors at baseline might have influenced the implementation and about sustainability over time. This resulted in a decision to perform another study, offering the tool to the remaining 32 PHC units in Östergötland County.

If there is an association between organizational climate and willingness to adopt a new way of working, climate could be seen as an implementation facilitator, and a climate assessment could help in developing a tailored implementation strategy. Organizational climate has earlier been found to be associated with youth outcomes in child welfare systems [12], but to our knowledge, no studies have compared organizational climate at baseline with a long-time perspective outcome of an implementation.

The aim of this study was to assess the association between organizational climate when a tool for lifestyle intervention was introduced in PHC and implementation outcome in terms of how the tool was perceived and used after 2 years.

## Methods

### Setting and study population

In 2008–2010, the CLT was offered to all 32 PHC units (i.e. health care centres with general practitioners (GPs) and other health care professionals) in Östergötland County, Sweden, that had not yet tried it. Of these, 22 units agreed to participate in the study.

### The CLT

The CLT consists of a lifestyle assessment performed on a touch-screen computer, and provides tailored advice to the patient, based on their individual answers. Staff members at the PHC unit are encouraged to refer their patients (aged ≥18 years) to the computer, and to give them the opportunity to discuss the results and provided advice. The CLT has been described in detail previously [7].

### Implementation strategy

The implementation strategy used was based on Rogers’ innovation–decision process including knowledge, persuasion, decision and implementation [5]. The implementation process began with an information session where a change agent from the research team visited the unit to provide information about the CLT. The information session was followed by a trial for 1 month, during which all staff members were encouraged to perform the lifestyle assessment provided by the CLT and give their opinions about it. The trial was part of the persuasion stage in Rogers’ model and the aim was to make the staff aware of the trialability and observability of the CLT. After the 1-month trial, the change agent visited the unit again. There was a discussion about how the CLT could be used within the daily routine, and mutual agreement to incorporate it as a working method or not was made. The purpose of this was to facilitate the decision stage. After the second meeting, the CLT was made available to patients.

### Data collection

Before the first visit at each unit, a questionnaire was sent to all staff members who meet patients in their daily work (administrative staff excluded) to assess the organizational climate. The Creative Climate Questionnaire (CCQ) developed by Ekvall was found to be feasible for the study [13]. The instrument measures individual perceptions of the organizational environment and has often been used in health care settings [13–16]. The CCQ instrument covers ten dimensions of organizational climate and consists of 50 statements that are answered on a 4-point scale (0–3) showing extent of agreement. The statements are put as: “There are many new ideas floating around here”. The ten dimensions are: Challenge, Freedom, Idea support, Trust/Openness, Dynamism/Liveliness, Playfulness/Humour, Debate, Conflicts, Risk taking and Idea time. The higher the value, the more creative the climate, except for the dimension Conflicts for which the reverse applies. Ekvall also presents reference values for innovative and stagnated organizations for each of the ten dimensions [13, 17].

When the CLT had been in operation for 24 months, another questionnaire was sent to the same staff categories. Respondents were regarded as a closed cohort, so that only those who had received the first questionnaire were included in the study population for the second questionnaire. As perceived need for the innovation has been found to be an important predictor for implementation [5], there was one question about this: “I see no need for the CLT”, to which the responder could agree or disagree. The other questions were formulated to capture how well the CLT had been adopted by the staff members. Some of the questions concerned how the CLT was perceived by the staff group, according to the responder, and are considered an evaluation of perceptions at the unit level; other questions concerned the individual’s personal perceptions of the CLT. One question concerned frequency of referral to the CLT, and is considered a measurement of use at the individual level. The questions and the response alternatives are given in the Results section.

### Data analysis

The CCQ assessment at baseline resulted in a mean value for each CCQ dimension, as suggested by Ekvall et al. [17]. Based on these results, the units were categorized into three groups, high CCQ (Hi), medium CCQ (Med) and low CCQ (Lo). The categorization was based on how many dimensions that, statistically significant, exceeded or fell short to the reference values presented by Ekvall et al. [13, 17] using the Student’s *t*-test. Units with a higher number of dimensions exceeding than falling short to the reference values were defined as Hi, units with less than six more dimensions falling short to than exceeding the reference values were defined as Med and units with at least six more dimensions falling short to than exceeding the reference values were defined as Lo. This resulted in 4 units in group Hi, 15 units in group Med,and 3 units in group low. See also Table 1. When the numbers were calculated the reverse nature of the dimension Conflicts was taken into account. The cut-off values were chosen to assure that units presenting top scores were compared to units presenting bottom scores.

**Table 1:** CCQ values in units defined as Hi or Lo and reference values for innovative organizations

Group (Hi or Lo)		Hi				Lo		
CCQ dimension	Reference value	Unit A (*n* = 15)	Unit Q (*n* = 10)	Unit U (*n* = 9)	Unit V (*n* = 16)	Unit K (*n* = 16)	Unit L (*n* = 15)	Unit M (*n* = 25)
Challenge	2.38	2.70^a^	2.24	2.47	2.41	2.11^a^	1.84^a^	1.81^a^
Freedom	2.10	2.13	1.93	2.10	1.93	1.53^a^	1.42^a^	1.55^a^
Idea support	1.83	2.37^a^	2.07	2.33	2.49^a^	1.38^a^	1.29^a^	1.45^a^
Trust/openness	1.78	2.29^a^	2.16^a^	2.47^a^	2.39^a^	1.71	1.22^a^	1.39^a^
Dynamism/liveliness	2.20	2.39	1.93	2.10	2.20	1.51^a^	1.04^a^	1.46^a^
Playfulness/humour	2.30	2.46	2.02	2.23	2.35	1.98^a^	1.31^a^	1.50^a^
Debates	1.58	2.17^a^	1.73	1.90	1.75	1.31^a^	0.98^a^	1.41
Conflicts	0.78	0.46^a^	0.18^a^	0.33	0.29^a^	0.75	1.20	0.78
Risk taking	1.95	2.04	1.69	1.73	1.83	1.35^a^	1.08^a^	1.26^a^
Idea time	1.48	1.79	1.47	1.60	1.50	0.98^a^	1.35^a^	0.89^a^

^a^Statistically significant difference from the reference value stated by Ekvall et al [16] as a threshold for an innovative organization

Associations between perceived need for the CLT and the CCQ dimensions were analysed with Spearman’s rank correlation coefficient. The Kruskall-Wallis test was used to calculate mean ranks regarding perceived need. Generalized Estimating Equations (GEE), which allows for analysis of clustered data, was used to calculate the Wald Chi-Square test in order to compare the distribution of responses to the follow-up questionnaire among the individual staff members from the three CCQ groups. Statistical analyses were performed using the computer-based analysis program, Statistical Package for the Social Sciences (SPSS) version 22.0. Statistical significance was set at p ≤ 0.05.

### Ethics

The study was carried out in compliance with the Helsinki Declaration and was approved by the Regional Ethical Review Board in Linköping, Sweden (Dnr Ö 16–08). As the participants were staff members, written consent was not required according to Swedish regulations (SFS 2003:460).

## Results

The average response rate was 53 % (range 30–81 %) at baseline and 52 % (range 20–86 %) at follow-up. The total number of respondents was 322 at baseline and 239 at follow-up. To be included at baseline, the responder had to have completed the CCQ instrument, leaving 275 (45 %) respondents for the analysis. An analysis of the drop-outs showed no differences between responders and the entire sample in terms of profession or gender, except that at baseline, the proportion of GPs was lower among the responders (12 %) than in the entire sample (20 %). CCQ scores for the units in group Hi and group Lo are shown in Table 1.

At follow-up, internal non-response occurred for some of the questions; the number of responders for each question is shown in Tables 2 and 3. The question on perceived need for the CLT revealed considerable differences between the units (*p* = 0.004). The mean ranks for each unit are presented in Table 4. When the item was analysed on a group level, groups Med and Lo showed homogeneity, with no significant differences within groups (*p* = 0.060, *p* = 0.808). Group Hi showed within-group differences (*p* = 0.003). It was found that the unit with the lowest perceived need was one of the units in the Hi group; this unit was excluded from further analysis, leaving three units in group Hi, which thus was homogeneous regarding perceived need (*p* = 0.481). The unit with lowest perceived need was also found to score low on perceptions and use. The question capturing use in terms of frequency of referral to the CLT showed significantly higher levels of use in group Hi compared with group Lo (p < 0.001) as shown in Table 2. For the follow-up questions assessing individual perceptions of the CLT, there was a significant difference between respondents in two of the three items when group Hi was compared with group Lo. Compared with staff in group Lo, staff in group Hi stated to a higher degree that the CLT facilitated their own work (*p* = 0.002) and that they believed the CLT could influence patients’ lifestyles (p < 0.001).

**Table 2:** Perceptions and use of the CLT related to the individual

Statement/question	CCQ group			Wald Chi-Square	*p* value
	Hi, % (*n)*	Med, % (*n)*	Lo, % (*n)*		Hi vs Lo
How often do you refer a patient to the CLT?				16.18	<0.001
Daily	0	1 (2)	0		
Once a week	35 (10)	28 (41)	21 (8)		
Once a month	52 (15)	43 (62)	49 (19)		
Never	14 (4)	28 (41)	31 (12)		
The CLT facilitates my work with lifestyle issues				9.95	0.002
Agree	11 (3)	10 (14)	6 (2)		
Partly agree	52 (14)	32 (43)	17 (6)		
Partly disagree	33 (9)	39 (52)	46 (16)		
Disagree	4 (1)	19 (25)	31 (11)		
It feels good/would feel good to refer patients to the CLT				3.27	0.071
Agree	46 (12)	29 (25)	20 (7)		
Partly agree	42 (11)	59 (51)	60 (21)		
Partly disagree	12 (3)	8 (7)	11 (4)		
Disagree	0	4 (3)	9 (3)		
It is my judgement that it is possible to influence patients’ lifestyles with the aid of the CLT				14.34	<0.001
Agree	35 (9)	16 (21)	6 (2)		
Partly agree	54 (14)	62 (83)	66 (23)		
Partly disagree	12 (3)	19 (25)	23 (8)		
Disagree	0	3 (4)	6 (2)

**Table 3:** Perceptions of the CLT related to the unit

Statement/question	CCQ group			Wald Chi-Square	*p* value
	Hi, % (*n)*	Med, % (*n)*	Lo, % (*n)*		Hi vs Lo
Staff often discuss the CLT				7.58	0.006
Agree	22 (6)	4 (5)	9 (3)		
Partly agree	52 (14)	27 (37)	23 (8)		
Partly disagree	22 (6)	45 (16)	49 (17)		
Disagree	4 (1)	24 (33)	20 (7)		
Using the CLT is well supported among the staff				19.42	<0.001
Agree	44 (12)	17 (23)	9 (3)		
Partly agree	48 (13)	48 (36)	43 (15)		
Partly disagree	7 (2)	25 (64)	43 (15)		
Disagree	0	10 (13)	6 (2)		
The CLT facilitates work with lifestyle issues at the unit				13.74	<0.001
Agree	15 (4)	11 (15)	6 (2)		
Partly agree	74 (20)	58 (78)	46 (16)		
Partly disagree	11 (3)	22(30)	43 (15)		
Disagree	0	9 (12)	6 (2)		
The CLT is today an important part of working with lifestyle issues at our PHC unit				10.78	0.001
Agree	30 (8)	15 (20)	9 (3)		
Partly agree	56 (15)	46 (61)	29 (10)		
Partly disagree	15 (4)	30 (40)	51 (18)		
Disagree	0	10 (13)	11 (4)

**Table 4:** CCQ group and perceived need for the CLT in terms of mean rank

Unit	Number	CCQ group	Mean rank^a^
I see no need for the CLT (agree/partly agree/ partly disagree/ disagree)			
A	10	Hi	55.65
B	12	Med	75.42
C	4	Med	78.75
D	4	Med	78.75
E	7	Med	79.36
F	4	Med	82.63
G	4	Med	82.63
H	8	Med	82.63
I	13	Med	83.19
J	7	Med	83.71
K	8	Lo	84.50
L	17	Lo	90.26
M	10	Lo	98.40
N	4	Med	100.38
O	6	Med	108.92
P	17	Med	115.71
Q	9	Hi	116.83
R	10	Med	121.30
S	19	Med	130.42
T	14	Med	136.79
U	8	Hi	139.88
V	9	Hi	142.28

^a^Mean rank according to the Kruskall–Wallis test for the units. Lower mean rank indicates less perceived need

For questions regarding perceptions at the unit level, group Hi was significantly more positive than group Lo for all four items assessed (Table 3). Among staff at the units in group Hi, the CLT was discussed more often (*p* = 0.006), use of the CLT was more supported (p < 0.001), was perceived to a higher degree to facilitate the work with lifestyle issues (p < 0.001), and was seen as an important part of the work with lifestyle issues (*p* = 0.001), compared with group Lo.

## Discussion

The aim of the study was to assess the association between the creative climate at baseline and perceptions and use of a tool for lifestyle intervention in PHC 2 years after its introduction. The main finding was that the units having a positive creative climate (group Hi) adopted the new tool to a higher degree than those with the least positive creative climate (group Lo). Differences were found regarding use at the individual level, and regarding perceptions at both the individual and unit levels. This result was based on self-reported data, and, at the individual level, concerned use in terms of frequency of referral, and perceptions of the CLT such as facilitating work with lifestyle issues and being an effective way to influence lifestyle. At the unit level, staff in group Hi were found to discuss the CLT, support its use and find that it facilitated work with lifestyle issues at the unit.

Staff in group Hi did refer patients to the CLT to a higher degree than staff in group Lo, but only one third of the respondents in group Hi referred patients more than once a month. This could not be considered a frequent use of the CLT. The fact that staff members express positive perceptions and seem to have adopted the new tool, but do not use it on a regular basis, could be explained in terms of conceptual or instrumental use, terms often applied in the context of research use [18]. Conceptual use refers to a change in the way practitioners think, their knowledge, understanding and attitudes, whereas instrumental use refers to the direct impact on practice decisions [18]. In the present study, staff at the units with a positive creative climate appreciate the CLT, which they find useful in their practice, but they still, after 2 years, have not incorporated it in their daily practice. In a former qualitative evaluation of the CLT at six PHC units, staff claimed that they often forget to refer their patients to the CLT, despite their good intentions [19]. This could also be due to the role of habit as a barrier for the implementation of change in clinical practice, which sometimes seems to be a stronger factor than knowledge and intentions [20]. The positive perceptions of the CLT found in group Hi, might result in a higher level of use in a longer perspective, but probably additional efforts from a change agent or the local management is still needed. Measuring creative climate at baseline could be one way to predict implementation outcome. It could be helpful in tailoring the interventions when an implementation process is planned, so that the implementation activities can be designed to suit the specific setting. A simplified way of describing tailored implementation is that it is similar to the tailoring of a clinical treatment to a diagnosed health problem [21]. Using that metaphor assessment of creative climate could help to diagnose the problem, and a tailored approach would be the treatment.

In our study the unit with the lowest level of perceived need showed high scores on creative climate, but low scores on perceptions and use of the innovation. Previous research has shown that a certain level of perceived need is important for an innovation to be successfully implemented [6]. If the potential adopters see no need for a certain new practice, it probably should not be implemented at all until staff have gained the required knowledge or curiosity about the innovation so that there is a so-called user pull as described by Lavis et al. [22]. When the implementation of the CLT was evaluated in another setting, in terms of sustainability after 2 years, staff expectations, perceptions of the innovation’s relative advantage and potential compatibility with existing routines were factors found to be positively associated with implementation outcome [23].

### Methodological considerations

Implementation is known to be a complex process, and there might have been other factors influencing the outcome, which could be seen as a limitation of the study. For example, adopter characteristics, outer context or the presence of opinion leaders, all potentially influencing factors according to Greenhalgh et al. [3], were not taken into account. Another limitation of the study is the use of self-reported data. It is known that self-reporting might be biased by social desirability and it cannot be ruled out that the respondents overestimated their perceptions and use of the CLT. This, however, could be assumed to be equal for all the participants. The sample size in this study is rather small, which may have influenced the GEE test so that the results are not as conservative as they should be, which is also a limitation.

Measuring creative climate using the CCQ is one way of assessing context. An advantage with the CCQ instrument is that it was originally developed in Sweden, and the disadvantages associated with translating, such as misinterpretation of questions, are avoided. The instrument has also been used and found feasible in other studies in Swedish health care settings [14–16]. Creative climate is known to be stable over time if no efforts are made to alter it [13], which is why no data on creative climate were collected at follow-up. Unexpected changes in creative climate might have affected the results.

The unit with the lowest perceived need and the highest creative climate was excluded from further analysis. A lack of perceived need at this stage, 2 years after the introduction of the CLT, was interpreted to mean that other ways of handling lifestyle issues were available, which made the CLT unnecessary and thus not used at all. Thus, the unit differed in such an important way from the other units in the CCQ Hi group that it was considered most appropriate to exclude it.

## Conclusions

According to the results from this study there is an association between organizational climate at baseline and implementation outcome after 2 years when a tool for lifestyle intervention is introduced in PHC in Sweden. Since the findings are based on self-reports and the use of the CLT was relatively low, further studies are needed before measurement of organizational climate at baseline can be recommended in order to predict implementation outcome.

## References

[1] Rycroft-Malone J, Kitson A, Harvey G, McCormack B, Seers K, Titchen A (2002). Ingredients for change: revisiting a conceptual framework. Qual Saf Health Care..

[2] Cummings GG, Estabrooks CA, Midodzi WK, Wallin L, Hayduk L (2007). Influence of organizational characteristics and context on research utilization. Nurs Res..

[3] Greenhalgh T, Donaldson L (2005). Diffusion of Innovations in Health Service Organisations: A Systematic Literature Review.

[4] Dopson S, FitzGerald L, Ferlie E, Gabbay J, Locock L (2002). No magic targets! Changing clinical practice to become more evidence based. Health Care Manage Rev..

[5] Rogers EM (2003). Diffusion of Innovations.

[6] Klein KJ, Sorra JS (1996). The challenge of innovation implementation. Acad Manage Rev.

[7] Carlfjord S, Nilsen P, Leijon M, Andersson A, Johansson K, Bendtsen P (2009). Computerized lifestyle intervention in routine primary health care: evaluation of usage on provider and responder levels. Patient Educ Couns..

[8] Health and Medical Services Act (1982:763) (in Swedish). http://www.riksdagen.se/sv/Dokument-Lagar/Lagar/Svenskforfattningssamling/Halso%2D-och-sjukvardslag-1982_sfs-1982-763/.

[9] Stange KC, Woolf SH, Gjeltema K (2002). One minute for prevention: the power of leveraging to fulfill the promise of health behavior counseling. Am J Prev Med..

[10] Geirsson M, Bendtsen P, Spak F (2005). Attitudes of Swedish general practitioners and nurses to working with lifestyle change, with special reference to alcohol consumption. Alcohol Alcohol..

[11] Johansson K, Akerlind I, Bendtsen P (2005). Under what circumstances are nurses willing to engage in brief alcohol interventions? A qualitative study from primary care in Sweden. Addict Behav..

[12] Glisson C, Green P (2011). Organizational climate, services, and outcomes in child welfare systems. Child Abuse Negl..

[13] Ekvall G (1996). Organizational climate for creativity and innovation. Eur J Work Organ Psychol..

[14] Bostrom AM, Wallin L, Nordstrom G (2007). Evidence-based practice and determinants of research use in elderly care in Sweden. J Eval Clin Pract..

[15] Norbergh KG, Hellzen O, Sandman PO, Asplun K (2002). The relationship between organizational climate and the content of daily life for people with dementia living in a group-dwelling. J Clin Nurs..

[16] Sellgren SF, Ekvall G, Tomson G (2008). Leadership behaviour of nurse managers in relation to job satisfaction and work climate. J Nurs Manage..

[17] Ekvall G (1990). Manual, Formulär A: Arbetsklimatet. (CCQ) [User’s Guide. Questionnaire A: Working Climate (CCQ)] (in Swedish).

[18] Nutley SM, Walter I, Davies HTO (2007). Using Evidence: How Research Can Inform Public Services.

[19] Carlfjord S, Lindberg M, Bendtsen P, Nilsen P, Andersson A (2010). Key factors influencing adoption of an innovation in primary health care: a qualitative study based on implementation theory. BMC Fam Pract..

[20] Nilsen P, Roback K, Brostrom A, Ellstrom PE (2012). Creatures of habit: accounting for the role of habit in implementation research on clinical behaviour change. Implement Sci..

[21] Wensing M, Bosch M, Grol R (2010). Developing and selecting interventions for translating knowledge to action. CMAJ..

[22] Lavis JN, Lomas J, Hamid M, Sewankambo NK (2006). Assessing country-level efforts to link research to action. Bull WHO..

[23] Carlfjord S, Lindberg M, Andersson A (2013). Sustained use of a tool for lifestyle intervention implemented in primary health care: a 2-year follow-up. J Eval Clin Pract..

